# An advanced prosthetic manufacturing framework for economic personalised ear prostheses

**DOI:** 10.1038/s41598-020-67945-z

**Published:** 2020-07-10

**Authors:** Rena L. J. Cruz, Maureen T. Ross, Jacob Skewes, Mark C. Allenby, Sean K. Powell, Maria A. Woodruff

**Affiliations:** Institute of Health and Biomedical Innovation, Queensland University of Technology, Brisbane, QLD Australia

**Keywords:** Health care, Quality of life

## Abstract

Craniofacial prostheses are commonly used to restore aesthetics for those suffering from malformed, damaged, or missing tissue. Traditional fabrication is costly, uncomfortable for the patient, and laborious; involving several hours of hand-crafting by a prosthetist, with the results highly dependent on their skill level. In this paper, we present an advanced manufacturing framework employing three-dimensional scanning, computer-aided design, and computer-aided manufacturing to efficiently fabricate patient-specific ear prostheses. Three-dimensional scans were taken of ears of six participants using a structured light scanner. These were processed using software to model the prostheses and 3-part negative moulds, which were fabricated on a low-cost desktop 3D printer, and cast with silicone to produce ear prostheses. The average cost was approximately $3 for consumables and $116 for 2 h of labour. An injection method with smoothed 3D printed ABS moulds was also developed at a cost of approximately $155 for consumables and labour. This contrasts with traditional hand-crafted prostheses which range from $2,000 to $7,000 and take around 14 to 15 h of labour. This advanced manufacturing framework provides potential for non-invasive, low cost, and high-accuracy alternative to current techniques, is easily translatable to other prostheses, and has potential for further cost reduction.

## Introduction

Microtia is a congenital malformation of the external ear that affects approximately 2 in 10,000 births^1^, and is diagnosed in varying degrees of deformity; from a slightly smaller ear to the complete absence of the external ear (known as anotia)^2^. Children with microtia and their families have been shown to suffer psychologically^3,4^ and use of a prosthetic or implantable solution can provide emotional relief^3,5^.

External ear prostheses are typically manufactured from medical grade silicone due to its skin-like appearance and mechanical properties^6–8^. The traditional hand-crafted fabrication process, however, is lengthy and retails $2,000–$7,000 per prosthesis^7,9^, though the manufacturing costs may be as low as $1,000^10^. A survey by Wiens et al. (1994) found that the average time to produce a prosthesis was 14 h 9 min^11^ while Mohammed et al. reported 15 h^12^. This process typically involves taking an alginate-gel or plaster impression of the contralateral ear if available (microtia is unilateral in 74–93% of cases^13–16^) to obtain patient specific ear morphology which can then be mirrored and the impression-taking process has been described as invasive and uncomfortable^12,17^. Next, the impression is used to make a positive plaster cast for use as a reference for carving a wax or clay prototype for the affected side. The prototype is tested on the patient and modified to improve the design. Finally, a mould is cast and silicone is set within the mould to produce the final prosthesis^6^. Despite the high cost, external ear prostheses typically only last around two years. This is due to damage which can occur, usually in the form of tears or discolouration^6^.

Advanced manufacturing (AM) techniques, including three-dimensional (3D) scanning, computer-aided design (CAD), and computer-aided manufacturing (CAM), can be collectively used to reduce the time, labour, and cost of prosthesis fabrication. 3D scanning may be used to non-invasively capture the patient’s unaffected ear, providing alternatives to uncomfortable alginate-gel/plaster impressions. Types of 3D scanning include expensive medical imaging (computed tomography (CT) and magnetic resonance imaging (MRI)^18–23^), in addition to lower cost optical surface scanning, techniques such as structured light scanning, laser scanning, and photogrammetry (3D photography)^24–33^. Non-portable medical imaging devices, such as MRI and X-ray radiating CT, can provide accurate and complete 3D ear morphology but are incredibly expensive to use and may involve exposure of the patient to X-rays. In contrast, lower-cost and portable optical surface scanning devices, are minimally invasive, non-ionising, and provide highly accurate 3D models of the patient’s anatomy at varying levels of completeness^24–33^. Scanning methods such as structured light scanning and photogrammetry represent the optimal combination of accuracy, time, cost, and invasiveness^28^.

Following scanning of the patient’s surface anatomy, 3D scan data is then processed using CAD to produce a 3D digital model suitable for 3D printing. Depending on the approach used, scan data can come in the form of 2D slice data^18–20^, several scans^10,25,26,33^, or 3D point clouds. The 3D data must then be processed using CAD software to create a complete 3D digital model. CAD software is also used for alignment of the digital prosthetic model with existing patient anatomy, removal of unwanted data, correction of inaccuracies from missing scan data, or incorporation of substructures^10,19,25,26,33^.

Computer-aided manufacturing (CAM) comprises both subtractive and additive (3D printing) processes able to automatically fabricate an ear prosthesis from a digital 3D CAD model. CAM has been used for the production of ear prosthesis prototypes, replacing the hand-sculpted prototypes^18,20,26,27^, and also used for fabricating moulds for silicone casting the final prostheses^10,19,25,30,33,34^. Progress is also being made to directly 3D print prostheses, removing the need for mould fabrication and manual silicone casting. One approach involves silicone infiltrated 3D printed starch prostheses which are commercially available for $4,000 each^35–38^. These prostheses, however, have inferior physical properties to typical silicone prostheses with faster degradation rates^37,39,40^. Direct silicone 3D printing technologies are currently in development by several groups. Approaches include the injection of catalyst into a vat of uncured silicone^41^, a dual-extrusion based system^42,43^, and a photocurable droplet deposition system^44^.

The most comprehensive additive manufacturing study for an ear prosthesis was a case study by De Crescenzio et al. (2010)^10^ which required a total of 10 h 20 min of labour to produce a prosthesis, a reduced labour time from the 14–15 h required to produce a typical handmade prosthesis^11,12^. In the present paper, we comprehensively describe an advance manufacturing framework to produce ear prostheses using current technologies, showing significant improvement from previous manufacturing frameworks and providing a benchmark on which further improvements can be implemented. This framework is also open to the incorporation of upcoming technologies including frugal scanning and model automation. Briefly, the framework involves 3D scanning the participant’s left ear, editing any scan defects to produce a complete ear model, designing a 3-part mould, printing the mould, and casting a silicone ear. This framework substantially reduces the labour, cost, and invasiveness involved, while maintaining a high degree of accuracy and resulting in a natural looking ear prosthesis.

## Results

Two approaches were studied. The first involved fabricating unsmoothed 3D printed polylactic acid (PLA) moulds and a straightforward silicone pouring technique to fabricate prosthetic ears matching six participants. The second approach trialled two improvements; (1) the use of solvent-surface-smoothed 3D printed acrylonitrile butadiene styrene (ABS) moulds and (2) a silicone injection casting technique. While the PLA moulds were fabricated on the LulzBot Taz 5 printer (Alech Objects, Inc., Loveland, CO, USA), the ABS moulds were printed on the Ultimaker 3 printer (Ultimaker B.V., Geldermalsen, Gelderland, Netherlands) with an enclosure kit prior to using the solvent vapour, acetone for surface smoothing. These differences in manufacturing led to corresponding differences in labour, cost, and aesthetic.

### Advanced manufacturing reduces production and labour time

The fabrication times for the two approaches are shown in Table 1, with times requiring input or supervision (i.e. contributing to labour time) highlighted in italics. The average time to produce a prosthesis using the unsmoothed 3D printed PLA moulds was 575 ± 52 min (9 h 35 min), of which the majority of the time was automated printing (59%) and an average “hands-on” labour time of 116 ± 11 min (1 h 56 min). The time to make a prosthetic ear using the acetone-smoothed 3D printed ABS moulds was longer, at 748 min (12 h 28 min) with 150 min (2 h 30 min) of labour. This additional time was mainly due to the setup requirements of the Ultimaker 3 printer and the time needed to smooth the ABS moulds to reduce the “staircasing” effect. This “staircasing” effect is an artefact of the layer-by-layer 3D printing process of desktop 3D printers where the individual layers are visibly noticeable and will transfer from the mould to the prosthesis.

**Table 1:** Mean times required for each stage of the ear prosthesis fabrication process when using 3D printed PLA moulds compared against a trail of smoothed 3D printed ABS moulds.

Process description	Time to produce unsmoothed AM prosthetic ears (min)	Time to produce smoothed AM prosthetic ears (min)
*3D scanning (two ears)*	*5 ± 3*	
*Scan processing*	*9 ± 2*	
*Prosthesis design*	*36 ± 9*	
*Scan alignment*	*7 ± 2*	
*Mould design*	*42 ± 13*	
*Print setup*	*6*	*20*
**3D printing of mould**	**339 ± 51**	**378 ± 51**
*Mould polishing set up and cleaning*	*–*	*20*
**Mould polishing**	**–**	**100**
*Casting*	*8 ± 1*	
**Silicone setting**	**120**	
*Demoulding and trimming*	*3 ± 1*	
**Total time**	**575 ± 82**	**748 ± 82**
*Total labour time*	*116 ± 31*	*150 ± 31*

Processes requiring human input are shown in italics, automatic/unattended processes are shown in bold.

### Advanced manufacturing is economical for high demand ear prostheses

The estimated setup and running costs for fabricating the prosthetic ears are summarised in Table 2. This table itemises the costs to produce 3D printed unsmoothed PLA mould-based prosthetic ears, and 3D printed ABS mould-based prosthetic ears with two further variations to the ABS method; (1) smoothed 3D printed ABS moulds with pour-casting, and (2) smoothed 3D printed ABS moulds with injection-casting.

**Table 2:** Table of approximate setup and running costs in US dollars.

	Cost item	3D printed PLA mould + poured silicone ears	Smoothed 3D printed ABS mould + poured silicone ears	Smoothed 3D printed ABS mould + injected silicone ears
Setup	Computer	2,000		
	3D scanner	23,000		
	Modelling software	4,000		
	3D printer	2,500	4,000	
	Smoothing device	–	10	
	Casting equipment	1,700		1,800
	Total	33,200	34,710	34,810
Running	Labour	116	150	
	Consumables	3	4	5
	Total	119	154	155

For more detail on items please see “Advanced manufacturing is economical for high demand ear prostheses”.

The table shows that the setup costs to produce unsmoothed 3D printed PLA mould silicone prosthetic ears, printed using the Lulzbot Taz 5 3D printer, is $ 33,200. Most of this cost is attributed to the high cost metrology rated hand-held scanner Artec Spider Scanner ($23,000). The use of the slightly more expensive Ultimaker 3 3D printer and enclosure kit adds approximately $1,500 to the setup cost, due to the need for a higher quality 3D printer capable of printing the ABS accurately. The cost of the smoothing device, which consists of a clear plastic chamber, is relatively insignificant at around $10. These total setup costs also include a computer for 3D modelling and to control the 3D printers of approximately $2,000, Cinema 4D modelling software to produce the digital mould designs of ~ $4,000, and silicone casting equipment of $1,700 for the silicone pouring approach and $1,800 for the silicone injection moulding approach. The casting equipment costs include digital scales, a vacuum chamber to remove bubbles from the curing silicone, and required tools such as a spatula and mechanical vice to hold the mould. The additional costs for the injection moulding approach includes labour and materials for the in-house manufacture of an injection moulding casing. As the setup costs are only needed to be paid once, the total costs of producing a prosthetic ear is largely dependent on the number of ears being produced with prosthetics able to be made very cheaply once the platform is set up.

The cost for consumables per prosthetic ear are relatively modest, as each ear only uses ~ 40 g of 3D printer filament and ~ 22 ml of PLATSIL GEL-0020 (prosthetic grade silicone), with costs of $27 per kg and $85 per litre, respectively. Including paper towels, plastic cups, petroleum jelly, a syringe and silicone pigment, the total costs for consumables are around $3 for the PLA mould based silicone prosthetic ear, and $4 and $5 for the poured silicone and injection moulded silicone ABS mould based prosthetic ears, respectively.

The most expensive component of the running costs for each silicone prosthetic ear is labour. Assuming a prosthetist salary of $80,000 per annum^45^ with 40% employer on-cost and 260 days worked per year and an average of 7.15 h of productive work per day (taking paid leave into account), the cost of employing a prosthetist is estimated to be approximately $ 60 per hour. This translates to around $116 of labour to produce a single PLA mould based prosthetic ear and $150 of labour to produce a single smoothed ABS mould based prosthetic ear, with the additional labour costs associated with the more complex 3D print job setup and ABS mould smoothing process.

Assuming that a traditional hand-made prosthesis will cost around $1,000 to manufacture^10^, significant cost savings are available using an AM approach as suggested in this study. Although the initial setup costs for AM approaches are relatively high compared to hand-crafted prosthetic fabrication, this is a once off fixed cost. For example, for a production run of 100 prosthetic ears the setup component of the per-unit cost is only approximately $330 for the PLA mould poured silicone prosthetic ear. Adding the labour cost in, the estimated cost per ear for 100 prostheses is around $450, less than half of the cost for a hand-made prosthetic ear.

### Advanced manufacturing produces highly accurate ear prostheses

**Figure 1:**
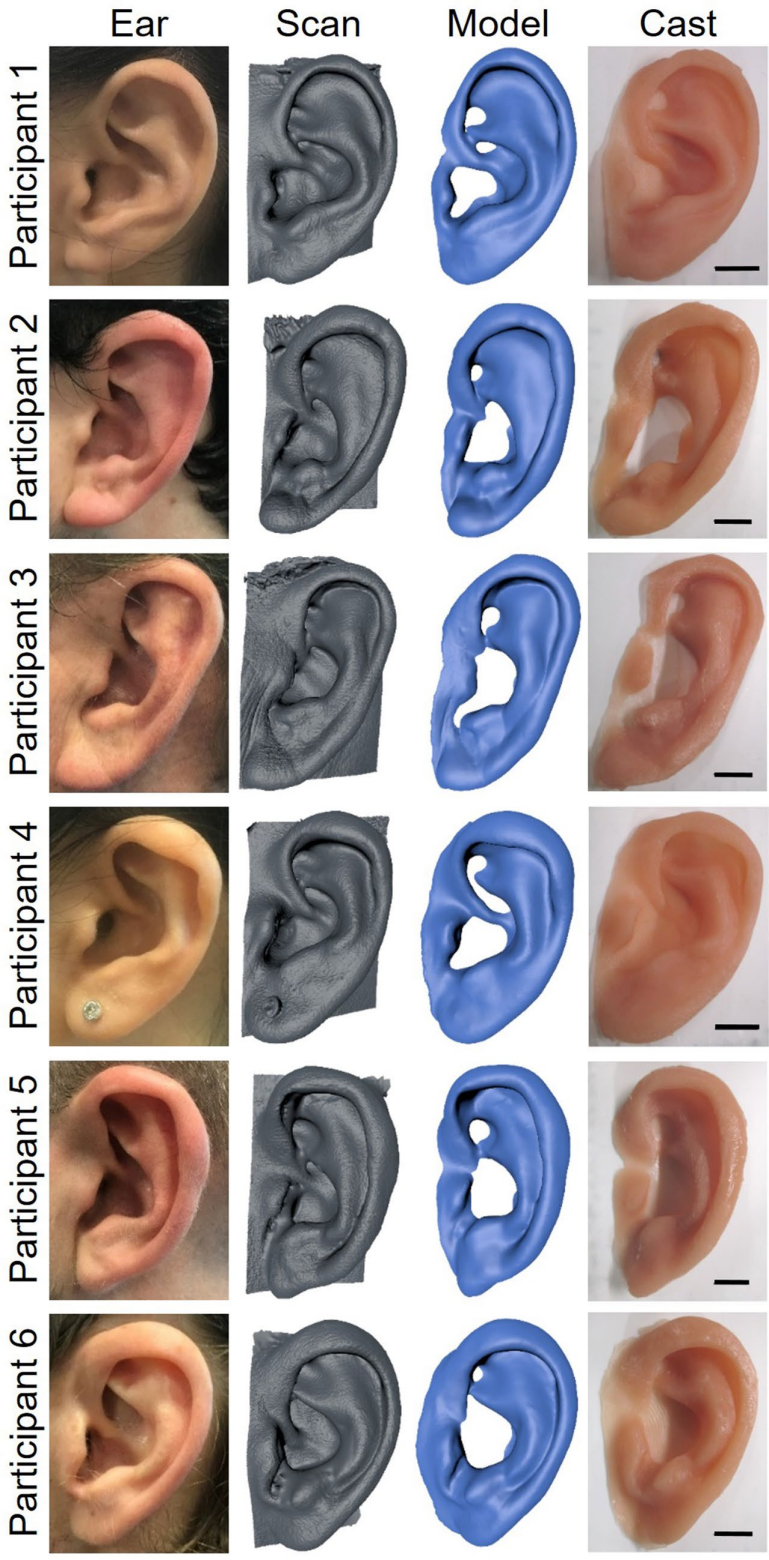
**(a)** Photographs of participants’ ears compared with 3D scans, 3D prosthesis models and silicone prostheses fabricated with unsmoothed 3D printed PLA mould approach with scale bar = 1 cm.

**Figure 2:**
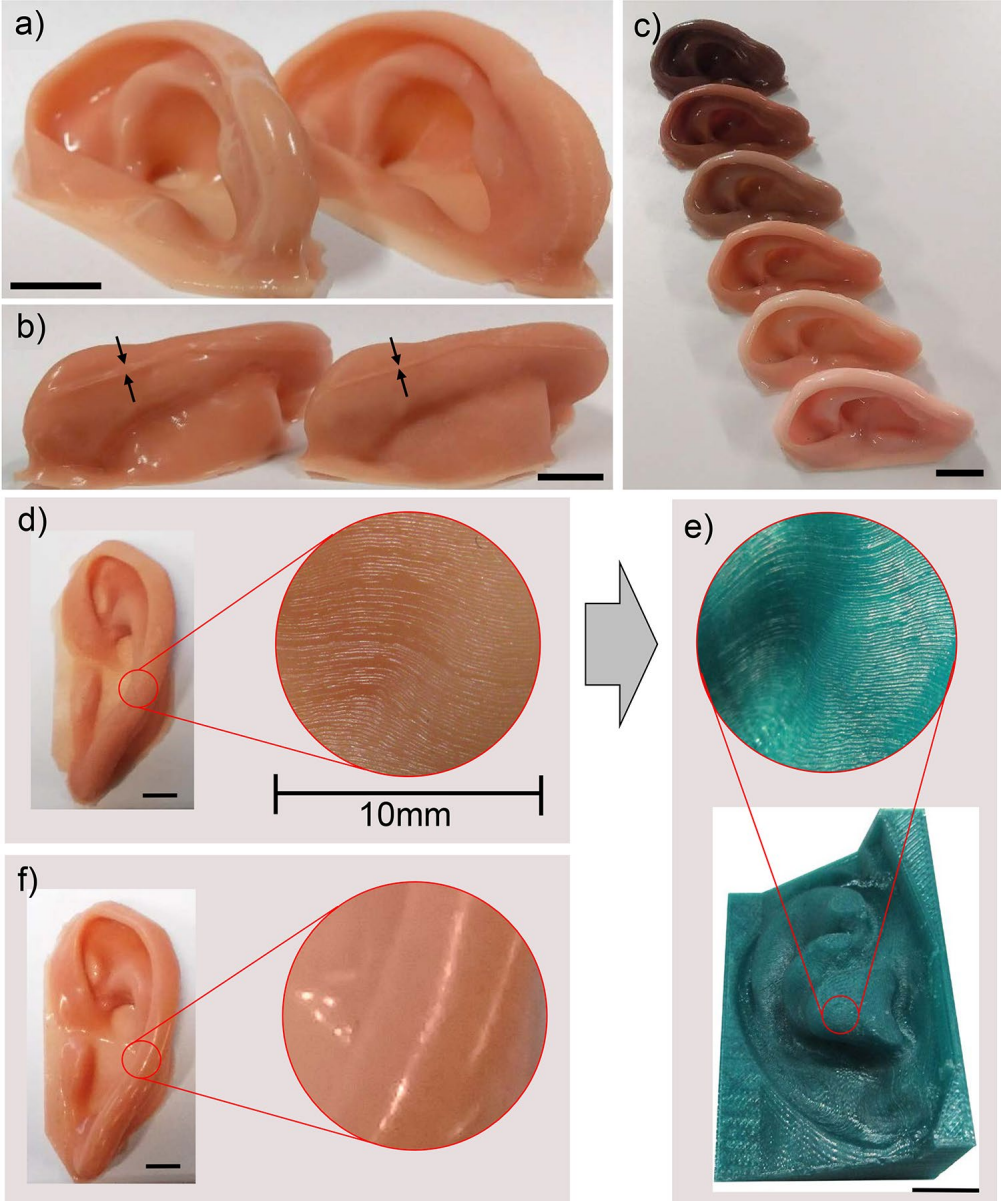
Photographs showing the effect of smoothing the ABS mould. **(a)** Front and **(b)** back views of prostheses made with the same mould before (right) and after (left) smoothing, and **(c)** several prostheses made with the smoothed mould in several skin shades. Black arrows depicting thickness of borders (flash) after and before smoothing. **(d)** Magnified view of the “staircase effect” on prosthesis showing **(e)** the “staircase” pattern on the 3D printed PLA mould. **(f)** Same ear showing how the smoothing removes this “staircase” effect. Scale bars = 10 mm.

Upon visual inspection of the ear prostheses made using the unsmoothed 3D printed PLA mould approach, there were no discernible morphological differences between the silicone ear prostheses and the original scanned ears. It is evident that an accurate ear anatomy was maintained through the entire process in comparison with the original ears, 3D scans, prosthesis designs, and final prosthesis (Fig. 1). However, a close inspection identified a micro-scale “staircase effect” on the surface of the silicone ears, imparted by an artefact on the moulds due to the layer-by-layer approach of 3D printing as shown in Fig. 2d and e.

To alleviate this artefact, an acetone vapour process was used to produce smoothed 3D printed ABS moulds, and furthermore, injection moulding was used to produce more consistent prosthetic ears by reducing bubbles. The effect of smoothing the mould prior to casting a prosthesis is clearly by comparing the prosthetic ears shown in Fig. 2a, b. Although the staircasing effect was removed, the smoothing of the sharp edges of the moulds resulted in thicker borders where the three pieces of the mould meet (known commonly as flash), seen clearly in Fig. 2b as indicated by the arrows. Figure 2d shows a more detailed look of the staircasing effect on a silicone prosthetic ear, which clearly corresponds to the layering pattern apparent in the 3D printed PLA mould shown in Fig. 2e. Figure 2f shows a magnified view of the same prosthetic ear produced using the smoothed 3D printed ABS mould. As the casting process is able to be easily repeated using the same mould, different combinations of silicone pigments can be added prior to casting producing silicone prosthetics of varying tone to match different patients as shown in Fig. 2c.

## Discussion

Advanced manufacturing (AM) provides a semi-automated approach to engineer customised products and is set to disrupt the current practices of medicine, where the automated production of highly personalised treatments will radically change existing practice, such as the production of prostheses. The traditional approach for prosthesis manufacture is very manual, expensive and labour-intensive, requiring 14–15 h^11,12^ of dedicated hand-crafting for an ear prosthesis. AM approaches, on the other hand, reduce the labour time required to manufacture ear prostheses. This will translate to positive impacts on many patients and clinicians, particularly as a typical clinic can fabricate over 70 ear prostheses per year^9^. This study describes an AM framework that reduces manual labour time and running costs by as much as 2.5 h and $150, respectively. This framework is significantly less laborious than many other published AM methods, and can be further developed to enable the automation of the computer design processes with the potential to further reduce manual labour^46–48^.

The most comprehensive, recent, publication describing AM ear prostheses was a case study by De Crescenzio et al. (2010)^10^; which required 27 h 20 min, including 10 h 20 min of labour. These times far exceed those of the present paper, even when accounting for the additional steps of substructure fabrication and extrinsic detailing. Importantly, the use of a stationary laser scanner in De Crescenzio et al.’s framework required the patient to remain still for 40 min followed by 6 h of CAD labour. This compares to a scan time of approximately 5 min, and 1 h 34 min for CAD processing (from scan processing to mould design) in our approach. A more recent study by Mohammed et al. (2018)^12^ chose, instead, to fabricate an ear prosthesis from CT scans by manually producing a mould from a moulded wax replica of a 3D printed ear model. This allows for the wax replica to be checked and edited for proper fit prior to producing the final mould. However, our methods found that a mould could have been directly modelled and printed with only an additional 55 min of labour and fabricated to be acetone-smoothed with an additional 1 h 29 min of labour instead, reducing labour while maintaining accuracy. The framework of Mohammed et al. would reduce a typical 15 h production time to 9–10 h, only reducing cost from $6,000 to $4,000. He et al. (2014)^30^ only specified that their simplified method required 2 h of labour. Other studies provided limited information regarding labour time^18,25–27,33,49^.

The setup cost of the approach with smoothed 3D printed ABS moulds with injection casting described in the present paper ($34,810) is significantly lower than the $200,000 setup cost reported for the AM methodology of Liacouras et al. (2011)^19^, which included a 3D photography system, software to compile CT scan data, CAD software, and a binder jetting (BJ) 3D printer. The study by He et al. (2014)^30^, which used low-cost equipment (Xbox Kinect sensor and $570 desktop printer), did not report the total setup costs and also produced a less detailed prosthesis compared to those of the present study. The highest cost component of our framework, the Artec Spider scanner, may not be a feasible purchase for many technicians, though a return on investment would occur at approximately 40 ear prostheses. This cost can be significantly reduced using lower cost scanning, such as photogrammetry with a smartphone, as proposed by Ross et al. (2018)^50^. There are also several free-to-use photogrammetry software packages available at no cost, however some of these require cloud storage which can pose privacy issues in a clinical context. The smartphone photogrammetry approach has additional benefits such as the ability for remote area and regional location scanning, improving equity of access. In addition smart phone cameras are becoming increasingly sophisticated with improved imaging sensors and imaging resolutions, and in some phones, include inbuilt depth sensing capabilities with potential for rapid and accurate 3D scanning of the patient’s ear. Additional cost savings can be made by using open-source and free-to-use 3D modelling software such as Blender (Blender Foundation, the Netherlands) in place of Cinema 4D which was used in the present study. Further up-front cost savings can be made by choosing a 3D printer that is lower cost than the $2,500 Lulzbot Taz 5 3D printer, although, care must be taken to ensure it is capable of consistent and reliable high quality results.

Despite the relatively high setup costs using our AM approach, the consumables costs are extremely low, with an average consumables cost of only around $ 4 per prosthesis. This amount is significantly lower than the corresponding material costs for AM (~ $120) approaches reported in the comprehensive publication by De Crescenzio et al. (2010)^10^, and in other publications describing AM methods^18,25,33^. The low cost in the present study can be attributed to the approach of in-house fabrication of the mould, with several other studies outsourcing the mould 3D printing to commercial suppliers^10,25^. Indeed, the present consumables costs were closer to the frugal printing methods of He et al. (2014)^30^ who reported a printing material cost of $0.80 from a total consumables cost of $8.60. However, the clinical application of their 1-part mould approach is comparatively limited due to difficulties producing a prosthetic ear that fits the unique anatomy of a patient’s face with only a single part mould. In our present approach it can be imagined that once an ear prosthetic is made, the mould can be retained by the provider and used again when a new prosthetic is required, substantially reducing the costs associated with future replacement prosthetic. However, the length of storage time for a given mould depends on both the environmental conditions of the storage location, such as temperature and humidity, as well as the chosen material of the mould. This is particularly important if the moulds are stored in locations of high humidity. PLA has been shown to be particularly sensitive to moisture and can often degrade more rapidly when not stored in dry environments, whereas, ABS is less susceptible to moisture related degradation^51^.

Comparing the aesthetic outcome of prostheses produced by AM methods, moulds fabricated with more expensive binder jet (BJ) printers showed superior surface quality^19,25^, without the presence of the staircase effect observed from desktop printers^30,33^. The staircase effect observed from desktop printers was minimised in the present study and the study by He et al. (2014)^30^ by printing an ABS mould and placing it in an acetone vapour environment prior to casting the silicone prosthesis. The acetone slightly dissolved and smoothed the surface of the mould which enabled the cast prosthesis to have a smoother surface. The 3D printed polylactic acid (PLA) moulds described in the present study cannot be easily smoothed, however, due to the lack of desirable chemical interaction between acetone and PLA. However, PLA is often preferred over ABS as it incurs significantly less polymer deformation during the cooling phase during 3D printing^52,53^. The cooling induced deformation apparent when 3D printing using ABS, however, can be prevented with the use of a fully enclosed printing system which insulates the printer and maintains a constant temperature. While smoothed 3D printed ABS moulds produced a smoother finish than unsmoothed PLA moulds in the final prosthetic ear (see Fig. 2), this came at the cost of thicker borders and increased setup, labour, and consumable costs. In fact, the aesthetic improvement is still under debate as the prosthetic ears took on a shinier appearance due to the smoothed mould and in fact may not be considered and aesthetic improvement by some patients, some level of topography may in fact provide a more realistic appearance.

CAM techniques have been shown to streamline the fabrication of an ear prosthesis compared to traditional hand-crafting approaches and are set to revolutionise the way patient-specific customised prostheses are made in the future. 3D scanning and computational modelling demonstrably reduce the labour required to produce moulds by replacing traditional hand-crafted approaches. Our framework demonstrates that, even accounting for additional extrinsic detailing, AM methods can significantly reduce labour time by up to 63% (including 3 h of detailing) and produce a high quality ear prosthesis compared to traditional hand-crafting approaches. These cost savings improve affordability and therefore patient accessibility to this treatment option. Although our advanced manufacturing framework has relatively high initial one-off equipment setup costs compared to traditional approaches, our analysis suggests these costs could be recuperated after the production of approximately 40 ears. Additionally, the nature of advanced manufacturing and 3D printing to easily produce different objects mean that these setup costs can also be incorporated in the production costs of other products and prostheses within a given clinic, for both hard and soft tissue prosthetics. Despite the high degrees of realism achievable with the computer aided manufacturing approach, including the ability to accurately match the patient’s intrinsic base pigment, the direct digital printing of extrinsic surface detail such as capillaries and fine colour variations is not yet available^54^. This additional step is traditionally performed through careful hand-painting by a prosthetist and, in some cases, can comprise a significant fraction of the total fabrication time and subsequently, cost. This fine detailing can provide extra realism for the patient which can be invaluable to supporting positive self-image for a patient. Although not yet developed, methods to automate this process using the high-resolution 3D texture information and digital printing has the potential to further reduce the time, and subsequent cost, of highly realistic prosthetic production. In the meantime, prosthetic production efficiency can be improved by streamlining the CAM production process to smoothly link to the hand detailing steps, including adding the ability for the prosthetist to reference the 3D scan model.

Finally, although superior to traditional approaches in both labour time and cost, the uptake of 3D printing in the prosthesis production industry is limited by the need for reskilling and training to operate the computer software and 3D printers. However, innovative solutions such as frugal 3D scanning using smart-phones, automated and purpose built computer modelling software, and more accessible and easy-to-use 3D printers can overcome these limitations and lead to the 3D printing of soft-tissue prostheses, such as prosthetic ears, becoming standard clinical practice.

## Methods

### Scanning participants

The sample cohort consisted of six adults; four Caucasian and two Asian participants from ages 24 to 51, none of whom had any discernible facial malformations. Institutional approval was obtained and all participants provided consent for image acquisition and scientific publication (QUT ethics approval number: 1600000770). All methods were performed in accordance with the relevant guidelines and regulations.

### Scanning the geometry of the ear

Scans were made of six participants’ left ears with an Artec Spider structured light scanner (Artec Group, Luxembourg). During scanning, participants were seated in an upright position and asked to remain still. Hair was kept back with hair pins and water to prevent any occlusion of the ear. Scan data was pre-processed into a mesh in Artec Studio 11 Professional (Artec Group, Luxembourg) to produce “watertight” (i.e. continuous) surfaces. The scans were saved in stereolithography (STL) 3D data format.

### Producing moulds for prosthesis fabrication

#### CAD reconstruction of ear scan geometry

**Figure 3:**
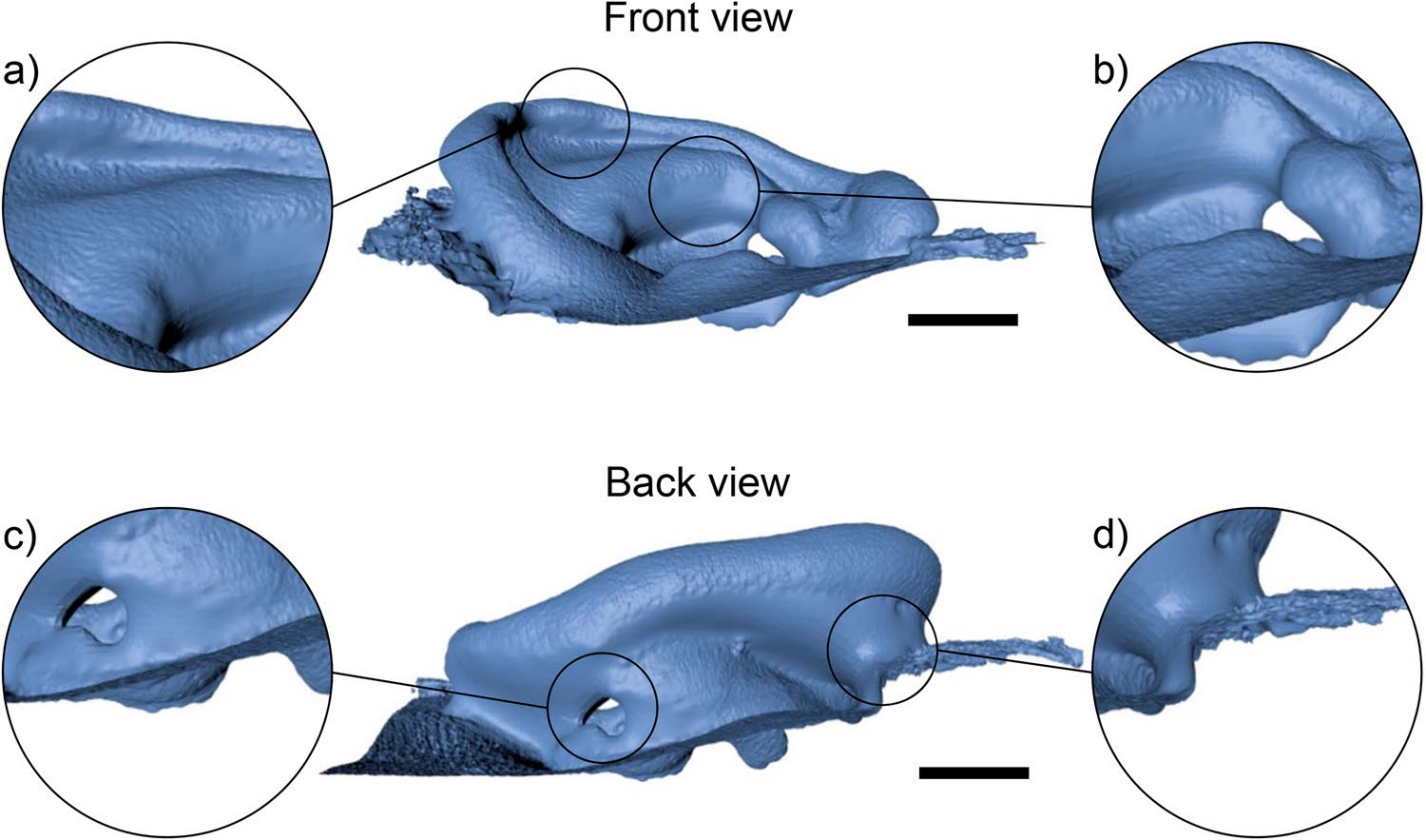
Examples of scan inaccuracies created by interpolation of missing scan data. Examples show interpolated areas **(a)** in the curve of the helix, **(b)** around the concha, **(c)** creating a hole through the ear and **(d)** behind the ear. Scale bar = 10 mm.

The scans were imported into the CAD software Cinema 4D R18 (MAXON Computer GmbH, Friedrichsdorf, Hesse, Germany), where inaccuracies such as holes (Fig. 3 c) and webs (Fig. 3 a, b, and d) were manually revised, typically taking less than 5 min. These inaccuracies were largely due to the automated creation of synthetic 3D data by the Artec Studio 11 software to remove excessive noise and interpolate missing data.

**Figure 4:**
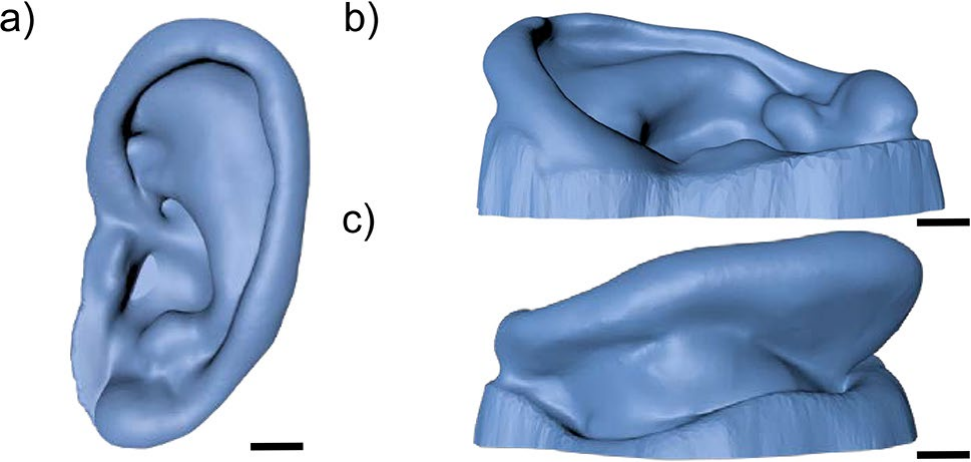
Ear prosthesis model and imitation microtic scan; **(a)** side, **(b)** front and **(c)** back view of example of final ear model. Scale bar = 10 mm.

Editing methods, such as the redefinition of the inner curve of the helix (Fig. 3a), removal of holes (Fig. 3c), and reconstruction of the back of the ear (Fig. 3d) to reflect the morphology of the front of the ear were undertaken. The models were cropped to isolate the ears and the surfaces rendered solid, depicted in Fig. 4, to produce the ear model.

#### Design of personalised ear prosthesis mould

**Figure 5:**
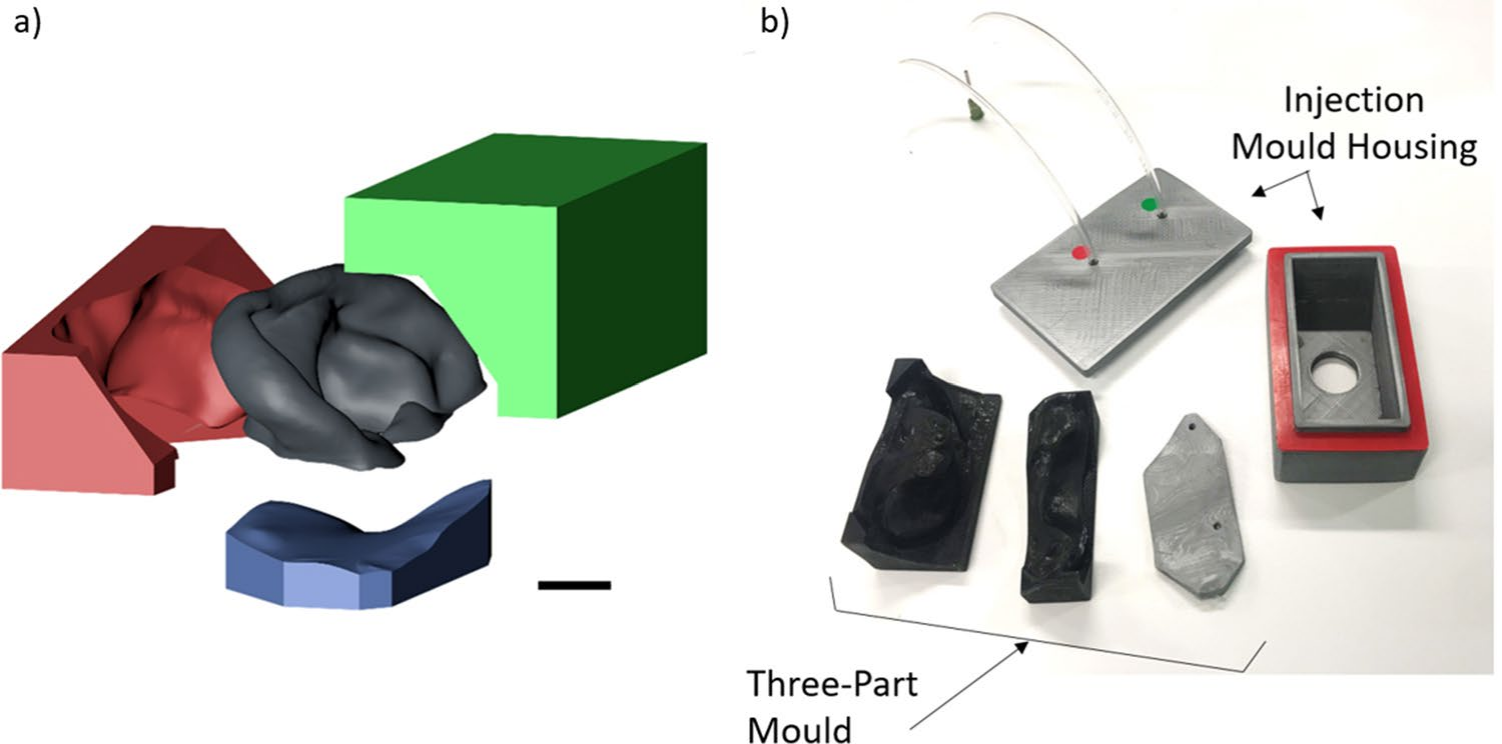
Exploded view of the 3-part mould with the prosthesis **(a)**; Block A in green, Block B in red, and Block C in blue; injection mould housing **(b)**. Scale bar = 10 mm.

The aligned ear models were further processed in Cinema 4D for the design of 3-part moulds and a universal injection moulding system shown in Fig. 5a and b respectively. Due to the complex shape of an ear, there is no orientation and removal direction which would permit the casted ear to be easily removed using a 2-part mould while achieving an accurate skin contact surface for prosthesis attachment. Thus a 3-part mould is necessary to avoid damaging the casted ear during de-moulding. The final 3-part mould layout was determined by considering aesthetic requirements, accuracy, 3D print-ability and ease of removal. As illustrated in Fig. 5a, the three parts of each mould were designed to reflect the three main surfaces of the ear prosthesis: (A) the outer surface of the ear, (B) the back surface of the ear, and (C) the skin surface of the prosthesis. This approach achieves a clean surface finish on the three main ear surfaces and places the parting line defect (caused by inaccurate surface mating’s of the mould parts) at the back of the ear in a less visible position. A 0.2 mm tolerance was incorporated between A and B. Similarly, a 0.6 mm tolerance was designed between A and C, and B and C. These tolerances were made to account for limitations in printing resolution. These three components were exported in the form of STL files for 3D printing.

#### Printing of 3-part mould

In an initial unsmoothed simple pouring method, parts A, B, and C of the mould were printed using PLA on a desktop fused-deposition-modelling (FDM) printer, the Lulzbot TAZ 5 (Aleph Objects, Inc., Loveland, CO, USA). Polylactic acid (PLA) (Filaform Australia, Adelaide) was used as the print material due to its relatively high-accuracy for low-stress applications. The layer height was set to 0.1 mm (setup in Lulzbot’s Cura 3D printing software).

A method of creating smooth moulds was trialled on one ear. Parts A, B and C of the mould were printed in acrylonitrile butadiene styrene (ABS) (Filaform Australia, Adelaide), a material whose surface could be smoothed in acetone. ABS has been known to deform during printing due to temperature changes. As such, these parts were printed in an Ultimaker 3 (Ultimaker BV, Netherlands) with an enclosure kit to maintain temperature. This 3D printer is a smaller device that can be insulated more effectively. The layer height was similarly set to 0.1 mm and any support material was removed after printing.

**Figure 6:**
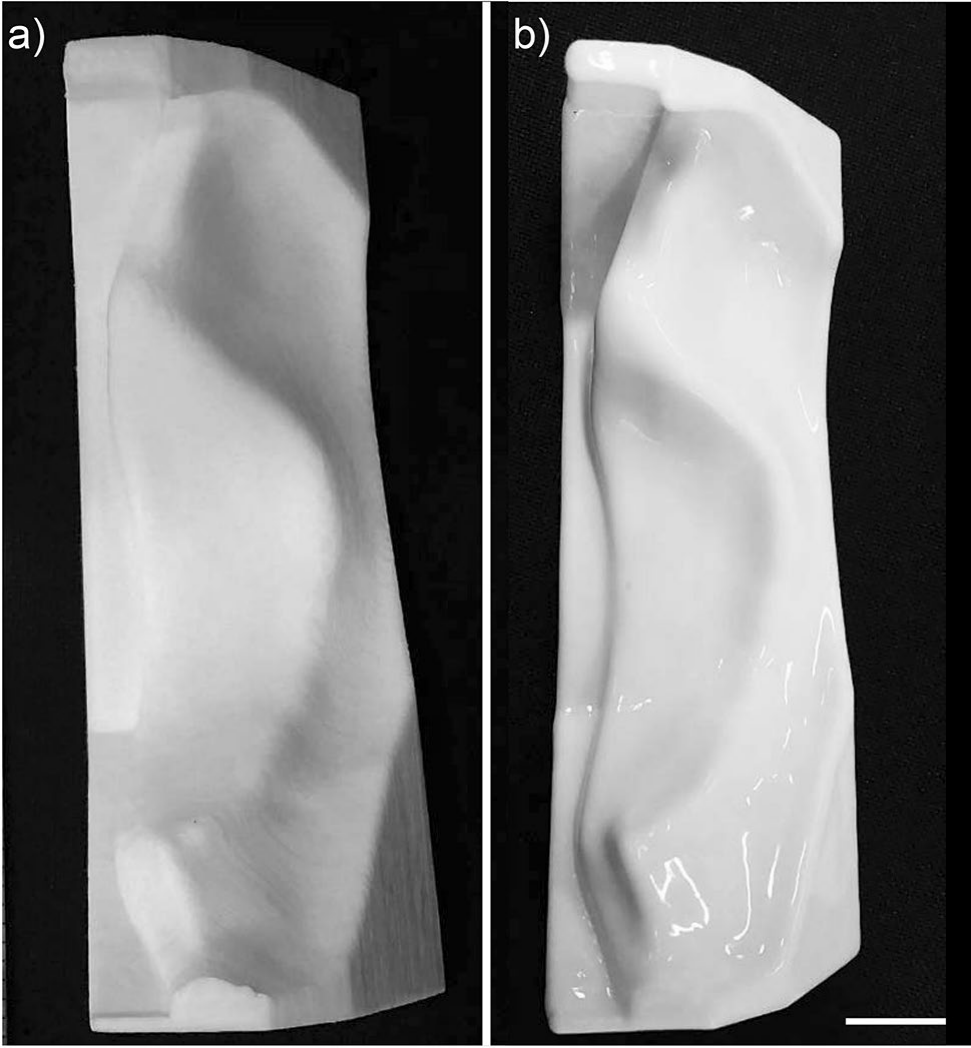
Part B of ABS mould **(a)** before and **(b)** after acetone vapour smoothing. Scale bar = 10 mm.

The mould parts were then surface treated using an acetone vapour smoothing process. The acetone vapour dissolves the surface of the mould, effectively blending the 3D printing layer lines together resulting in a smooth shiny finish as demonstrated in Fig. 6.

This process was completed by first loosely scrunching 9 Kimtech Kimwipes and placing them in the base of a large glass bowl, then wetting the Kimwipes with 25 ml of acetone at room temperature. The purpose of the Kimwipes is to act as a carrier for the acetone and increase the surface area contact between the surrounding air and acetone to allow the rate of evaporation to occur more quickly and evenly around the 3D printed mould. A steel block was placed into the centre of the glass bowl to be used as a stand for the 3D printed mould, keeping it from coming into direct contact with liquid acetone. The bowl was then covered with a glass lid to keep the acetone vapour from escaping and treated for 50 min, at that time the parts begin to look slightly ‘wet’. Finally, the 3D printed mould was left to dry for a further 50 min as Acetone dissolves the ABS material deep enough for the smoothing effect to continue after removal.

### Prosthesis fabrication

#### Casting the prosthesis

PLATSIL GEL-0020 (Polytek Development Corp, Easton, PA, USA), a prosthetic-grade two-component (1:1) room temperature vulcanising (RTV) addition-cured silicone elastomer was prepared. This preparation involved mixing the two components in equal quantities with of 0.2% (g/ml) “Flesh” pigment (measured on scientific scales) and degassing the mixture in a vacuum chamber to remove bubbles.

For the pouring method, the silicone mixture was poured into parts A and B which were secured together in a vice. Part C was then used as a cap to enclose the uncured silicone. After 2 h, the mould was removed and any excess silicone (flash) trimmed.

On occasion, air pockets were noted in the resultant prostheses from enclosing the mould. A more sophisticated injection method was devised and tested. To create the prosthesis, all three mould parts were assembled and placed into a 3D printed enclosure that had inlet and outlet holes. The silicone mixture was loaded into a syringe and injected into the assembly through the inlet until the silicone was seen exiting through the outlet (see Fig. 5b). After 2 h, the mould was removed, and excess silicone trimmed. Additionally, one ear mould was used to cast several identical ears in different skin tones to display the rage of skin tones that can be achieved by mixing different percentages (g/ml) of silicone pigments of flesh, black, white, red, and yellow to the two components of the silicone elastomer prior to degassing to achieved the desired skin tone

#### Methods comparison

Labour, expense, and aesthetics of this AM framework was compared with traditional ear prosthesis fabrication. The durations for each stage of the fabrication process for each of the six prostheses fabricated with the unsmoothed 3D printed PLA moulds were recorded and averaged and compared with a single trial using smoothed 3D printed ABS moulds. Set up and ongoing costs were calculated using available cost data and aesthetics were visually compared with original ears. The equipment and materials were purchased in Australian currency and have been converted to US dollars for comparison (1 AUD = 0.70 USD, August 2019).
